# Highlighting Bacteria with Calcifying Abilities Suitable to Improve Mortar Properties

**DOI:** 10.3390/ma15207259

**Published:** 2022-10-17

**Authors:** Iuliana Răut, Mariana Constantin, Ionela Petre, Monica Raduly, Nicoleta Radu, Ana-Maria Gurban, Mihaela Doni, Elvira Alexandrescu, Cristi-Andi Nicolae, Luiza Jecu

**Affiliations:** 1 National Institute for Research & Development in Chemistry and Petrochemistry-ICECHIM, 202 Independentei Splai, 060021 Bucharest, Romania; 2 Faculty of Pharmacy, Titu Maiorescu University, 16 Bd. Gheorghe Sincai, 040441 Bucharest, Romania; 3 CEPROCIM S.A., 6 Preciziei Street, 062203 Bucharest, Romania; 4 Faculty of Biotechnology, University of Agronomic Sciences and Veterinary Medicine of Bucharest, 59 Mărăşti Boulevard, 011464 Bucharest, Romania

**Keywords:** *Bacillus*, concrete, biomineralization, ureolytic bacteria

## Abstract

Biomineralization, the use of microorganisms to produce calcium carbonate, became a green solution for application in construction materials to improve their strength and durability. The calcifying abilities of several bacteria were investigated by culturing on a medium with urea and calcium ions. The characterization of the precipitates from bacterial cultures was performed using X-ray diffraction, Fourier transform infrared spectroscopy, and thermogravimetric analysis. The formation of carbonate crystals was demonstrated by optical and scanning electron microscopy. Water absorption and compressive strength measurements were applied to mortars embedded with sporal suspension. The efficiency of the supplementation of mortar mixtures with bacterial cells was evaluated by properties, namely the compressive strength and the water absorption, which are in a relationship of direct dependence, the increase in compressive strength implying the decrease in water absorption. The results showed that *Bacillus subtilis* was the best-performing bacterium, its introduction into the mortar producing an increase in compressive strength by 11.81% and 9.50%, and a decrease in water absorption by 11.79% and 10.94%, after 28 and 56 days of curing, respectively, as compared to standards. The exploitation of *B. subtilis* as a calcifying agent can be an interesting prospect in construction materials.

## 1. Introduction

Concrete is the most widely used construction material due to its durability, high compressive strength, and long-term performance. It is a concern but also a challenge to improve the overall quality of cementitious materials by extending the life of structures and reducing the maintenance costs. The development of construction materials is an important sign of technological progress. On the one hand, the increasing need of concrete, and on the other hand, the possible degradation and damage produced by natural environments have led to innovations through the use of technological bioengineering. Nowadays, it is considered that the traditional methods for cement repair are limited due to cost and complexity, and therefore, the application of biomineralization has received great attention for its potential as alternative solution to increase the durability of cement [1,2,3,4].

Microbially induced calcium carbonate precipitation (MICP) is a natural biomineralization process based on activity of specific microorganisms able to induce the precipitation of calcium carbonate (CaCO_3_). The biomineralization plays an important role in the cementation of natural systems (soil, caves, etc.), and it can occur through following mechanisms, such as: urea hydrolysis, photosynthesis, sulfate reduction, denitrification, ammonification, and methane oxidation. Of these mechanisms, the metabolic pathways of ureolytic bacteria have been examined most extensively due to the simple functional principle in urea hydrolysis catalyzed by secreted urease [5,6,7,8]. The biomineralization through urease mechanism can be simplified as follows: bacterial cells which are negative charged adsorb calcium ions from the environmental solution (calcium chloride, calcium acetate, calcium lactate, and calcium gluconate); urea added is hydrolyzed by bacterial urease producing CO_3_^−2^ and NH_4_^+^; CO_3_^−2^ combines with Ca^2+^ to precipitate calcium carbonate crystals; finally, the bacterial cells are surrounded by carbonate crystals. The rate of mineralization is directly related to urea hydrolysis. Accordingly, the ureolytic mechanism producing calcium carbonate has been adopted for several applications in construction, as bio-cement [9,10,11,12,13,14], environmental protection including the remediation of heavy metals and radionuclides [15,16,17], CO_2_ sequestration [18], soil and sand consolidation [19,20,21], and other applications, such as materials engineering [22].

In last few years, there has been a growing interest in the use of microbially precipitated CaCO_3_ as biomaterials, and more studies have been published [7,23,24,25,26,27].

The main factors that can impact microbially induced carbonate precipitation are bacterial genotype and cell concentration, appropriate amounts of Ca^2+^ (supplemented externally) and urea (for CO_3_^2−^ production), nutritional composition of medium for bacteria cultivation, and pH conditions for urease activity [28,29,30]. Likewise, the availability of crystal nucleation sites was found to greatly affect the calcium attachment, and finally, the formation of calcium carbonate crystals [31], although “the mechanism that controls the growth and crystallization process remains unclear and controversial” [32].

Recently, the known microbial mineralizing bacteria used for MICP were reviewed [33,34]. *Sporosarcina pasteurii*, previously known as *Bacillus pasteurii*, is the most used bacterium for studying microbially induced calcium carbonate precipitation due to its high urease activity. Gradually, different other bacteria have been studied for inducing carbonate precipitation under various laboratory conditions, mainly *Bacillus* species including *B. megaterium*, *B. subtilis*, *B. cereus*, *B. licheniformis*, *B. mucilaginous*, cyanobacteria such as *Synechocystis* sp. and *Synechococcus* sp., nitrate-reducing bacteria *Pseudomonas aeruginosa*. The two kinds of bacteria, *Bacillus* and *Sporosarcina*, present strong adaptability to the environment, high specific surface area of cells, and they can use urea as energy and nitrogen source in metabolism. Therefore, careful considerations are required when choosing bacteria because the type of bacteria influences the morphology and deposition rate of calcium carbonate [34].

Hence, the objectives of the present study were: (i) to evidence the calcifying abilities of several bacteria strains expressed under proper culture conditions, and (ii) to study the effect of microbial-induced calcite precipitation for improving the physical–mechanical properties of cement mortar. For this purpose, four different microbial strains from the Microbial Collection of National Institute for R&D in Chemistry and Petrochemistry- ICECHIM were cultivated on medium with urea and calcium ions.

## 2. Materials and Methods

### 2.1. Microorganisms and Culture Conditions for Testing Carbonate Precipitation with Alizarine Red S

Four bacterial strains, as *Bacillus *amyloliquefaciens 1014** and *Bacillus licheniformis* 1015 from the Microbial Collection of National Institute for R&D in Chemistry and Petrochemistry-ICECHIM (Bucharest, Romania)*,* *Bacillus subtilis* ATTC 6633*,* and *Pseudomonas putida* LMG 2257 (purchased from DSMZ, German Collection of Microorganisms and Cell Cultures GmbH, Braunschweig, Germany) were employed in tests for the ability to precipitate calcium carbonate. The bacterial strains were cultured on Nutrient Broth (NB) (Scharlau, Scharlab, Barcelona, Spain) medium and Luria Bertani medium supplemented with urea and calcium chloride (Table 1). The mixture was autoclaved at 121 °C for 20 min, and then urea and calcium chloride were sterilized separately by filtering through a 0.2 µm syringe. The final concentrations were 20 g/L for urea and 25 g/L for calcium chloride, respectively. The experiments were carried out in 100 mL Erlenmeyer flasks, incubated at 30 °C and 100 rpm for 3 days. A 0.10% solution of alizarin red S was used as an indicator for carbonate precipitation. Alizarin powder was dissolved under energic agitation in approx. 80 mL distilled water, and pH was corrected at 4.0 with HCl. Then, the solution was completed at 100 mL, filtered through a 0.22 µm syringe filter and kept at 4 °C in the dark. The culture broths were filtered, and the filter paper was sprayed with alizarin red S solution prepared as above. The appearance of pink color was considered a positive result.

### 2.2. Qualitative Test for Ureolytic Activity

The strains were activated by cultivation in Tryptic Soy Broth (TSB, Difco Laboratories, Detroit, MI, USA) (Table 1). Bacterial strains were cultured on various agar and liquid selective nutrient media for screening the ureolytic activity (Table 1). Urease selective medium without urea was employed as the control medium. Corresponding solid medium was prepared by the addition of 1.5% agar. The positive result of test as the hydrolysis of urea was considered the change of color from yellow/orange to pink, which occurred after 48 h of incubation at 37 °C [37,38].

### 2.3. Cultivation of Bacterial Strains for Calcium Carbonate Precipitate

The strains were cultured on TSB medium to allow the growth and sporulation, and then, on medium with urea and calcium ions to facilitate carbonate precipitation (Table 1). After sterilization, the medium for carbonate precipitation was amended with 28 g/L CaCl_2_ and 20 g/L urea (sterilized by filtering through 0.2 µm syringe) and then incubated in an orbital shaker (110 rpm), 30 °C for 8 days [39,40]. After incubation, the culture broths were filtrated, and the pellets were dried overnight at 50 °C and analyzed through various techniques, such as SEM, FTIR, TGA, and XRD.

### 2.4. Microscopical Observations

Morphological analysis of samples was carried on an Olympus BX 51 microscope (Tokyo, Japan) and Quanta FEI 200 Scanning Electron Microscope (SEM, Brno, Czech Republic).

### 2.5. Analysis of Precipitates from Bacterial Cultures

FTIR measurements of the samples were performed using a Spectrometer JASCO FT-IR 6300 (Oklahoma City, OK, USA) with accessory Pike GladiATR-single reflection Attenuated Total Reflectance mode (Fitchburg, WI, USA). All samples were recorded in the wavelength range of 4000–400 cm^–1^, averages of 64 scans per spectrum, with the resolution of 4 cm^−1^.

Thermogravimetric analysis (TGA) was performed using a TGA Analyzer Q5000IR (TA Instruments, New Castle, DE, USA), and the following conditions were applied: 22~32 mg of samples, nitrogen (99.999%) 50 mL/min, ramp 10 °C/min to 700 °C, RT = 25–40 °C (Room Temperature), sample pan. Platinum 100 μL, T_max_ (°C) = T(dα/dt)_max_.

The precipitation from cultures was collected and analyzed by X-ray diffraction using a Rigaku SmartLab 9 kW diffractometer (Tokyo, Japan), in 2*θ*/*θ* configuration, between 10 and 90° (2θ) [41,42]. Samples from culture without urea and calcium ions were used as controls.

### 2.6. Preparation of Bacterial Mortars Prisms

*B. licheniformis* and *B. subtilis* were used for the preparation of mortars prisms. The pre inoculum was prepared in physiological serum from fresh bacterial stock culture. The suspension was inoculated on Luria Bertani medium supplemented with agro-industrial by-product and then incubated at 28–30 °C for 24 h. The by-product had the role of supporting the viability of bacterial spores. The details regarding the preparation of sporal suspension are not presented because they are the subject to a patent application under evaluation [43]. The sporal suspension thus obtained was used in tests, replacing the water in the mortars. Two sets of prisms were made: (i) conventional mortar prisms mixed with tap water (standard), (ii) mortar prisms mixed with 1.0 optical density of bacterial sporal suspension, measured by spectrophotometer BioMate (Thermo Fisher, Waltham, MA, USA) at wavelength 600 nm (OD_600_ = 1.0). The mortars were prepared using a cement/sand ratio of 1:3 by weight and water/cement ratio of 0.5. The mortar mixes were obtained on automatic mortar mixer (ToniMIX, Berlin, Germany) and then casted into molds (40 × 40 × 160 mm) and compacted on a vibration machine. After 24 h, the sample mortars were demolded and cured in solution of 0.33 M urea and 0.025 M calcium chloride (water for standard) at 20 ± 2 °C until the test terms. Broken samples collected from mortar prisms after 2, 7, 28 and 56 days of curing were used for further analysis, namely water absorption and compressive strength.

### 2.7. Investigations of Mortar Samples

Water absorption measurements were carried out after 2, 7, 28 and 56 days of curing following the procedure described by ASTM C642-97 [44]. The water absorption is calculated from the following equation:

$$A=\frac{W_{2}-W_{1}}{W_{1}}\cdot 100$$

where A = water absorption, %

W_1_ = mass of oven dried sample in air, g
W_2_ = saturated mass of sample after immersion, g

The compressive strength of cement mortars was determined after 2, 7, 28 and 56 days of hardening following the procedure described by SR EN 196-1 [45] using a Form + Test Seidner compression machine with maximum capacity 250 kN, accuracy class 1 and automation rate of load increase of 2400 N/s.

## 3. Results

### 3.1. Screening of Microorganisms and Culture Conditions

**Figure 1:**
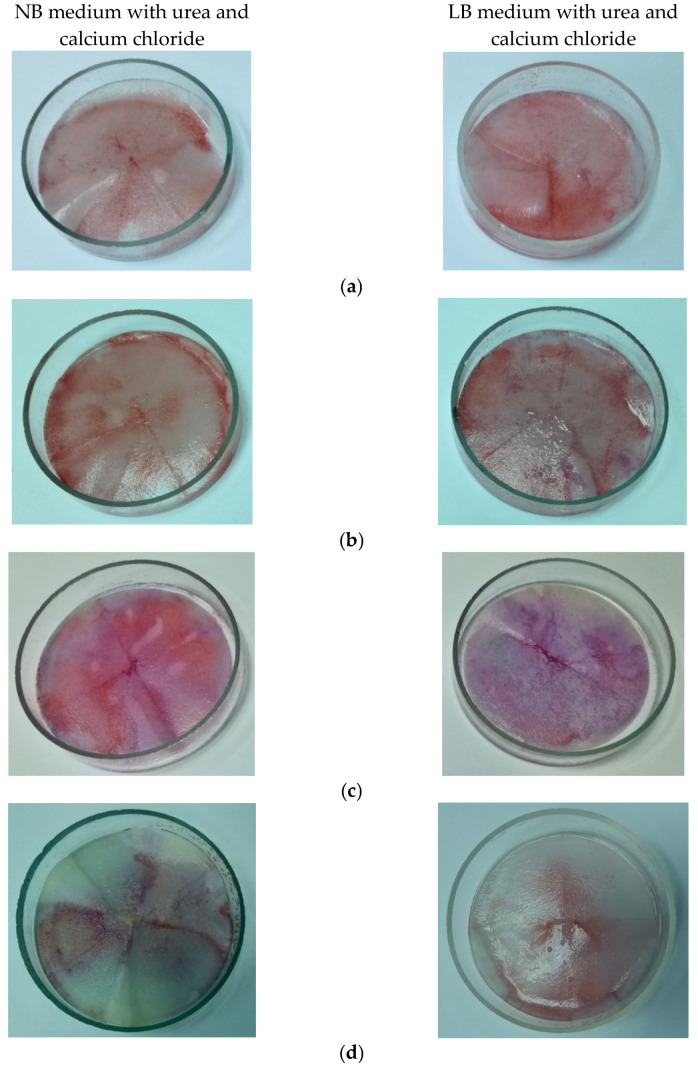
Alizarin colorimetric assay for testing carbonatogenesis ability of bacterial strains: (**a**) *Bacillus* *amyloliquefaciens*; (**b**) *Bacillus licheniformis*; (**c**) *Bacillus subtilis*; (**d**) *Psuedomonas putida*.

The experimental tests were carried out with four bacteria strains belonging to *Bacillus* and *Pseudomomas* genera. *Bacillus subtilis, Bacillus amyloliquefaciens* and *Bacillus licheniformis* are Gram-positive bacteria, forming endospores that allow them to survive for a long period of time in extreme conditions. Bacillus species grow by forming chains of cells and then a thin layer, which is an important behavior to fill voids in the mortar matrix. *Pseudomonas putida* is a Gram-negative, non-spore-forming saprotrophic bacterium, having a hard cell wall which makes it more resistant than Gram-positive bacteria. These bacteria can be easily controlled and are widely available in the soil and natural environment. Bacterial strains were cultivated on media supplemented with urea and calcium chloride to sustain the precipitation of calcium carbonate since the biomineralization is based on ureolytic mechanism [11,46,47]. The results of a colorimetric test with alizarin red S applied to bacterial strains are presented in Figure 1. Alizarin red S Is a common anthraquinone dye used in histology and histopathology to stain calcium carbonate deposits. The alizarin color turns progressively from pale yellow (pH = 5.2) to pale rose (pH = 7.1) and then to violet (pH = 10.1), and in about 3–5 min, it intensified, as it can be seen. In the test, we observed that the red–violet color as a positive result exposed by *Bacillus* strains was more evident in comparison with those from *Pseudomonas*, which exhibited a lower level of biomineralization. *Bacillus subtilis* showed an intense violet color.

Taking into account the results, the following experiments were carried out only with all three *Bacillus* strains which present more important characteristics. The spores of Gram-positive *Bacillus* could better resist the mechanical and chemical stress during the mortar preparation. In addition, Gram-positive bacteria have thicker cells compared to Gram-negative bacteria, which sustain their viability in pores of the mortar matrix.

**Figure 2:**
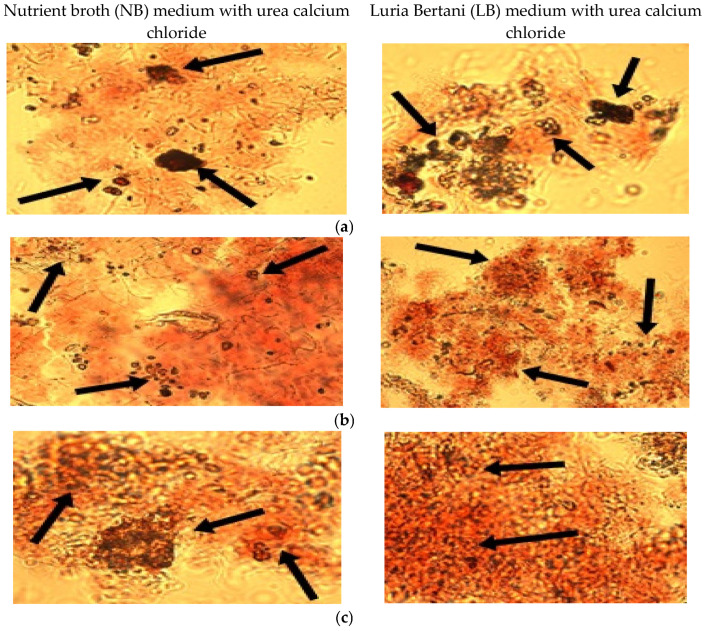
Optical microscopy applied to bacterial culture broth after coloring with red alizarin S (×40): (**a**) *Bacillus* *amyloliquefaciens;* (**b**) *Bacillus subtilis;* (**c**) *Bacillus licheniformis.* Black arrow—calcium carbonate crystals.

The culture broth was filtered through filter paper Whatman 1, and microscopical observations of culture deposits were made. The presence of calcium carbonate crystals inside bacterial biomass was evidenced (Figure 2).

### 3.2. Qualitative Test for Ureolytic Activity

**Figure 3:**
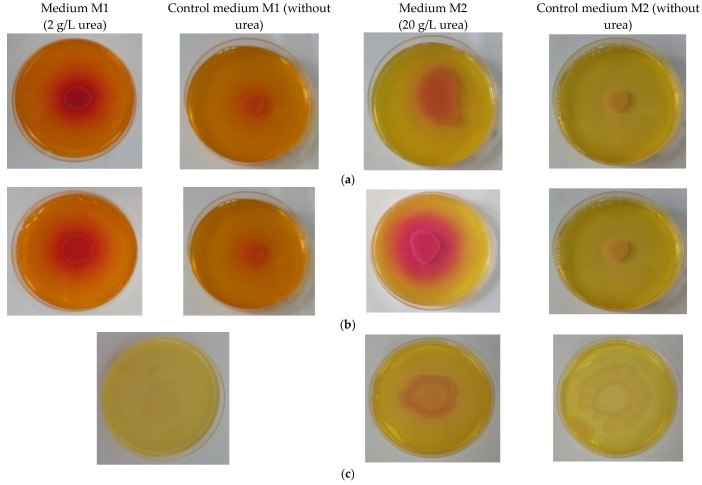
Ureolytic activity of bacterial strains in Petri plates on solid media of different compositions: (**a**) *B. licheniformis* on media M1, M2 and their corresponding controls (without urea and calcium ions)*;* (**b**) *B. subtilis* on media M1, M2 and their corresponding controls (without urea and calcium ions)*;* (**c**) *B. amyloliquefaciens* on media M1, M2 and their corresponding controls (without urea and calcium ions).

The *Bacillus* strains were cultured on various agar-selective nutrient media for screening the ureolytic activity. The positive test result of urea hydrolysis was considered the changing of medium color into pink red around the inoculation point with bacterial culture, which is due to the expression of urease enzymatic activity. The results are presented in Figure 3. *B. amyloliquefaciens* had no activity on medium M1 with a small amount of urea (2 g/L), while *B. subtilis* and *B. licheniformis* exhibited a moderate amount of activity. The slightly reddish color that appeared in plates with control medium M1 was probably due to the large amount of phenol red from the culture medium. On medium M2 with a higher amount of urea, *B. subtilis* showed a good ureolytic activity, which was followed by *B. licheniformis* and *B. amyloliquefaciens*. No results were obtained on medium M3 with NiCl_2_ (data not shown).

### 3.3. Cultivation of Bacterial Strains for Calcium Carbonate Precipitate

**Figure 4:**
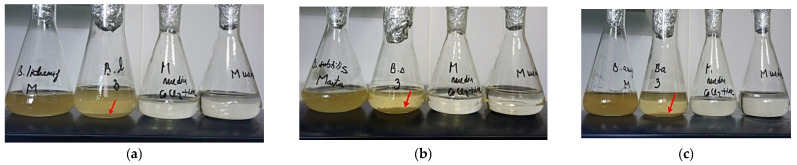
Images of experiments performed with *Bacillus* strains in medium with or without urea and Ca^2+^: (**a**) *B. licheniformis;* (**b**) *B. subtilis;* (**c**) *B. amyloliquefaciens.* In order from left to right, bacterial culture on TSB medium; bacterial culture on medium with urea and Ca^2+^; M_medium_, control, as medium mineral with urea and Ca^2+^, non-inoculated; M_medium_, control as medium mineral without urea and Ca^2+^, non-inoculated; red arrow—white deposit containing bacterial biomass and calcium carbonate.

To evidence the precipitation of calcium carbonate, *Bacillus* strains were cultured under orbital agitation on nutrient broth medium amended with urea and calcium chloride (Figure 4). The white precipitates appeared almost instantly at the bottom of the conical flasks, and its density increased with incubation.

**Figure 5:**
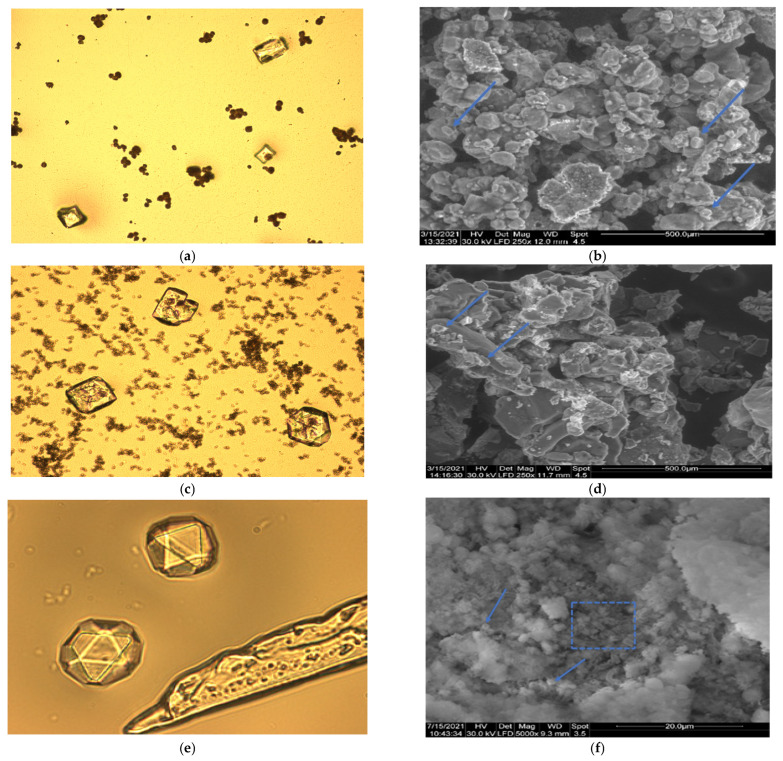
Microscopic investigations of deposits obtained from bacteria cultured on medium with urea and calcium ions. On left, optical microscopy is shown, and on the right, SEM analysis, as: (**a**) Spores and crystals carbonate from *Bacillus amyloliquefaciens* (20×); (**b**) SEM analysis of pellet from *Bacillus amyloliquefaciens* ^(^250×); (**c**) Spores and crystals carbonate from *Bacillus licheniformis* (20×); (**d**) SEM analysis of pellet from *Bacillus licheniformis* (250×); (**e**) Crystals carbonate and germens of crystallization at *Bacillus subtilis* (100×); (**f**) SEM analysis of deposit from *Bacillus subtilis* (5000×). Dotted square-bacterial cells; arrow—calcium carbonate crystals.

The microscopic observations of deposits from bacterial cultures, mixtures of calcium carbonate precipitate and bacterial biomass, are presented in Figure 5.

The optical images clearly highlight the crystals of calcium carbonate (Figure 5a,c,e). The SEM captures (Figure 5b,d,f) showed calcified bacterial cells covered by mucous matrix, which makes it difficult to highlight images of crystals. In Figure 5f, an agglomeration of bacterial cells at a magnitude of 5000× can be observed.

### 3.4. FTIR Analysis

**Figure 6:**
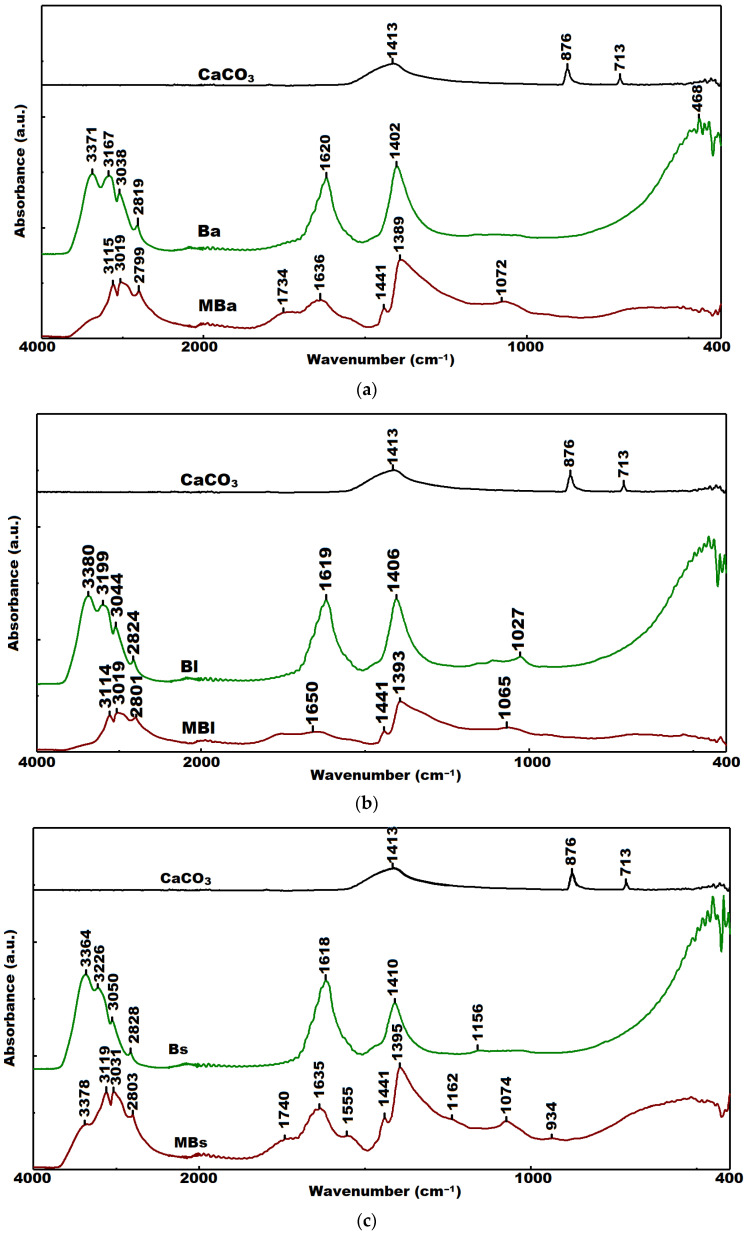
Overlaps of FTIR spectra of bacterial deposits from cultures on media with or without urea and calcium ions: (**a**) *Bacillus amyloliquefaciens* (**b**) *Bacillus licheniformis*; (**c**) *Bacillus subtilis*.

The precipitates were studied by FTIR spectroscopy (Figure 6).

In our experimental study, a broad vibrational band corresponding to the O-H stretch of the carboxyl group at 2700–3300 cm^−1^ was identified for all samples. However, at the same time, within this domain, it is possible to have a band assignment to NH_2_ stretching in adenine, cytosine or guanine [48]. In addition, we have detected a vibrational band at 1620–1650 cm^−1^ assigned to the amide-I, indicating the presence of proteins in the structure of the precipitates. The planar asymmetric stretching band at 1400–1413 cm^−1^ assigned to carbonate was identified in samples form bacteria cultivated in medium with urea and Ca^2+^. The bands assigned to the phosphoric ester group P-O-C were identified at 1020–1070 cm^−1^, confirming the presence of phosphate-containing components in the structure of the precipitates. The bacterial activity leads to the formation of extracellular polymeric substances (EPSs), mainly including polysaccharides, proteins, glycoproteins, nucleic acids, and lipids. Only in precipitates of control samples from *B. subtilis* and *B. amyloliquefaciens,* we identified an absorption band of C-O stretching at 1734–1739 cm^−1^ which could be attributed to a lactam of muramic acid that belongs to characteristic structures of endospores [48].

### 3.5. TGA Analysis

**Figure 7:**
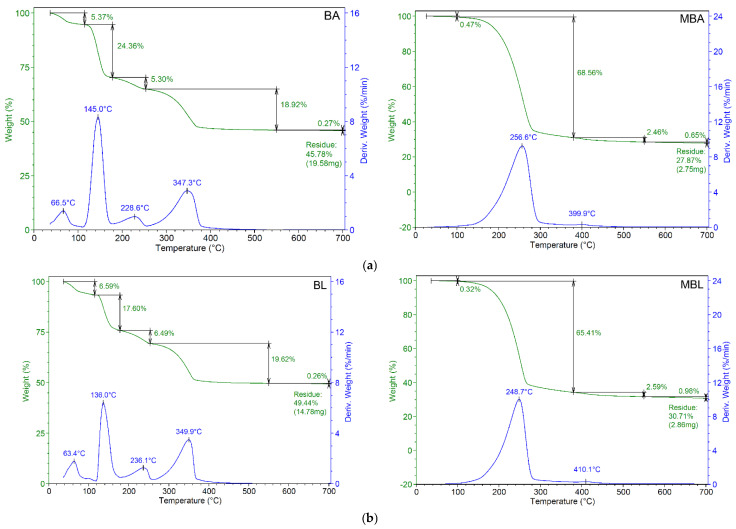

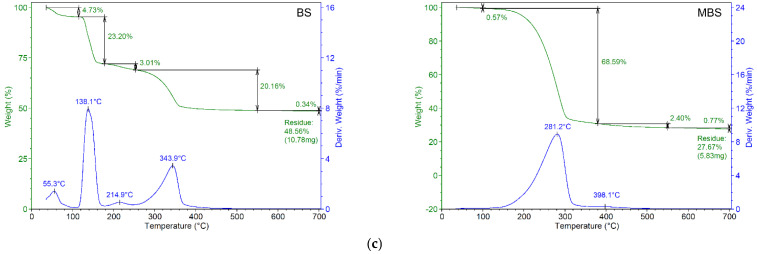
TGA curves of precipitates obtained from bacterial cultures on medium with or without urea and Ca^2+^: (**a**) TGA curve of precipitates obtained from *B. amyloliquefaciens* cultured on medium with (BA, left) or without urea and Ca^2+^ (MBA, right); (**b**) TGA curve of precipitates obtained from *B. licheniformis* cultured on medium with (BL, left) without urea and Ca^2+^ (MBL, right); (**c**) TGA curve of precipitates obtained from *B. subtilis* cultured on medium with (BS, left) or without urea and Ca^2+^ (MBS, right).

The thermogravimetric analysis of precipitates from bacterial cultures on medium with or without urea and Ca^2+^ is presented in Figure 7 and Table 2 and Table 3.

The deposits from bacterial culture on medium with urea and Ca^2+^ were characterized by the occurrence of three main weight loss steps, at 115–178 °C, 253.5–550 °C, and over 700 °C. In contrast, the TGA of precipitates from control samples presented only two main weight loss steps, at approximately 250–280 °C and over 700 °C, their behavior being different as compared with samples from cultivation on medium with urea and Ca^2+^. During the TG analysis, the samples exhibited a first slight weight loss in the range of RT–110 °C, representing approx. 4–7%, higher than those obtained for control samples, approx. 0.3–0.5%.

The first main weight loss for all samples occurred at the temperature domain of 115–178 °C (24.36%; 17.60%; 23.20% for *B amyloliquefaciens, B. licheniformis* and *B. subtilis*, respectively) and at 253.5–550 °C (18.92%, 19.62, and 20.16% for *B amyloliquefaciens, B. liqueniformis* and *B. subtilis*, respectively) (Table 1). In the first temperature domain, *B. amyloliquefaciens* had the highest value of weight loss of 24.36% (138 °C), while *B. subtilis* had the highest weight loss of 20.16% in the second temperature range of 253.5–550 °C. The weight loss that occurred in the temperature range of 253.5–550 °C was caused by the release of structural water and associated with the pyrolysis of organic or inorganic macromolecules. Lipids, proteins and other volatile organic compounds are decomposed in this temperature region [49].

Referring to control samples (Table 2), the main loss of moisture takes place between 100 and 380 °C, being the main active pyrolysis region. The percentages of weight loss are decreasing in the following order: 68.59%, 68.56% for *B. subtilis* and *B. amyloliquefaciens*, respectively, >65.41% for *B. licheniformis*.

The most significant result is connected to residue percentage. After exposure at temperature over 700 °C, the residues percentages of samples from bacterial cultivation in media with urea and Ca^2+^ ions (average values of 45–50%) are significantly higher than those obtained from control samples (average values of 27–30%). This behavior is explained by the presence of precipitated CaCO_3_ mixed with bacterial biomass. The values of residue percentages are decreasing in the following order: 49.44% for *B. licheniformis* > 48.56% for *B. subtilis* > 45.78 for *B. amyloliquefaciens.* The corresponding data for control samples are decreasing in the following order: 30.71% for *B. licheniformis >* 27.87% for *B. amyloliquefaciens >* 27.67% for *B. subtilis.*

### 3.6. XRD Analysis

**Figure 8:**
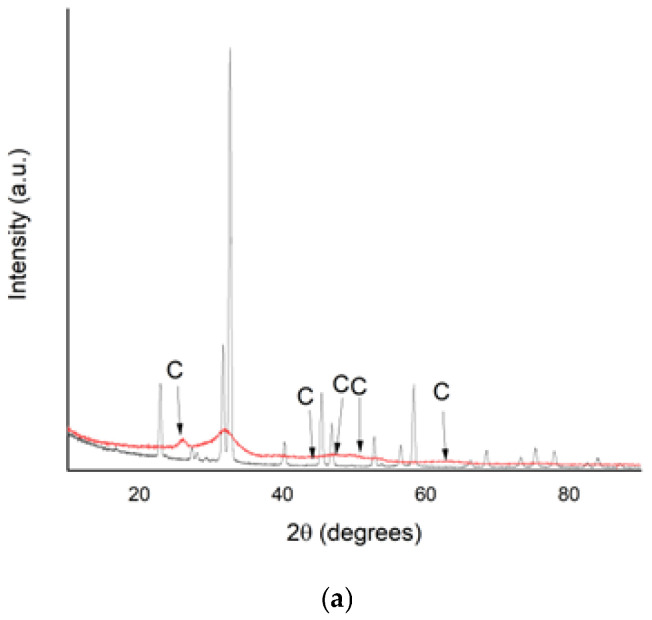

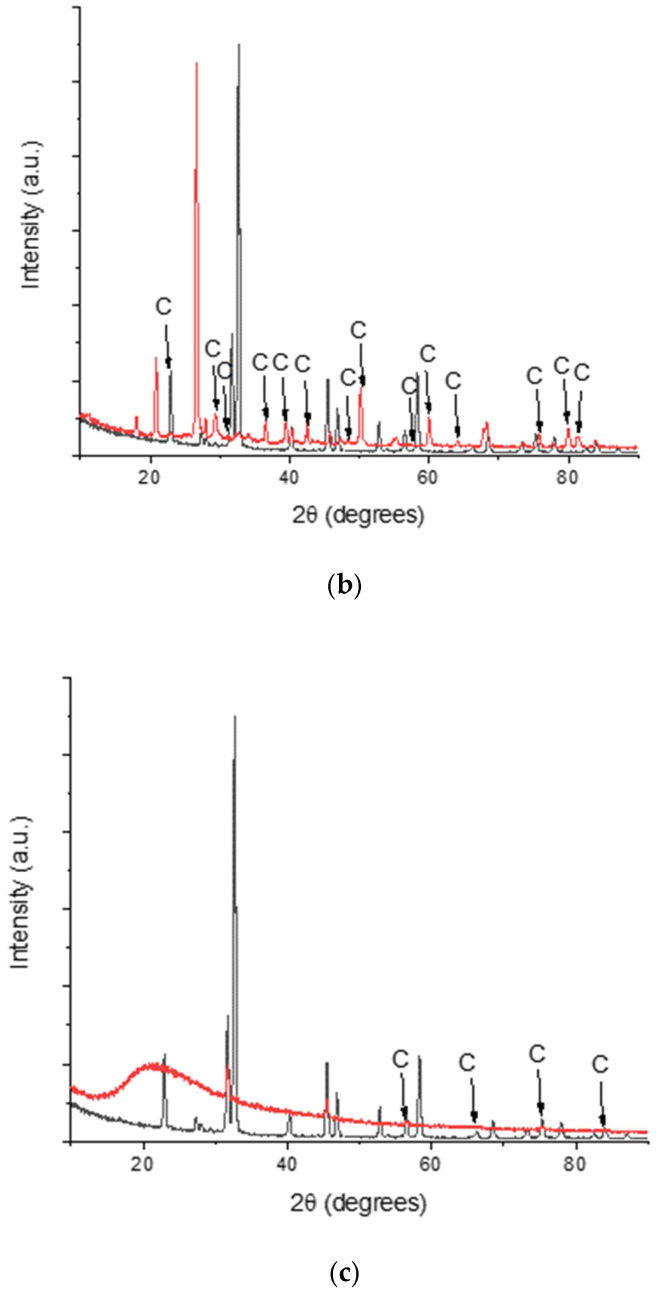
XRD patterns of deposits recovered from bacterial cultured on medium with or without urea and Ca^2+^ ions: (**a**) *Bacillus licheniformis;* (**b**) *Bacillus subtilis;* (**c**) *Bacillus amyloliquefaciens* (red line—sample; black line—control, medium without urea and Ca^2+^ ions; C—calcium carbonate as calcite).

Figure 8 displays the XRD pattern of deposits from bacterial cultures on media with or without urea and calcium chloride. The distinct diffraction peaks as calcite occurred at different positions, as follows (2θ): at 27°, 44°, 46°, 52° and 62° for *Bacillus licheniformis*, at 12°, 22°, 26°, 30°, 32°, 40°, 42°, 46°, 48°, 50°, 58°, 64°, 68°, 76°, and 80° for *B. subtilis*, and 22°, 58°, 62°, 64°, and 80° for *Bacillus amyloliquefaciens*. No peaks of vaterite were noticed in our samples. The calcite peaks were identified by reference to the ICDD entry 01-083-0577, without considering its possible secondary crystallization phases in relation to the support structure. These secondary crystallization processes led to the formation of new crystalline (Figure 8b) or predominantly amorphous (Figure 8c, with the presence of some minor peaks that can be attributed to the calcium carbonate phase) structures.

### 3.7. Compressive Strength and Water Absorption

**Figure 9:**
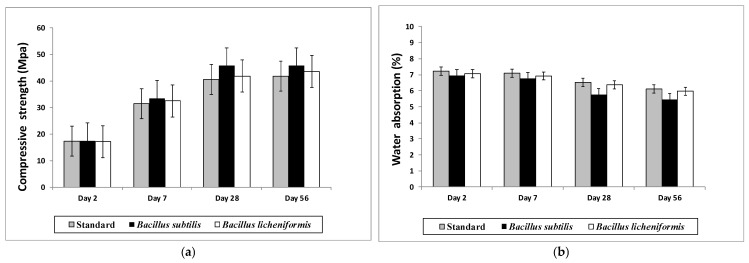
Compressive strength (**a**) and Water absorption (**b**) of mortar specimens after 2, 7, 28 and 56 days of curing.

The results of tests for compressive strength and water absorption are presented in Figure 9. The mortar specimens noted that *B. subtilis* and *B. licheniformis* contained bacterial cells and were cured in solution of 0.33 M urea and 0.025 M calcium chloride at 20 ± 2 °C until the test terms. Mortar standards did not contain bacterial cells and were cured in water. All experiments were performed at least three times, and the results are shown as standard deviation.

The compressive strength of mortars after 2, 7, 28 and, respectively, 56 days of curing (Figure 9a) showed the positive effect exerted by the introduction of bacteria cells from *B. subtilis*. Thus, the increase was low at samples after 2 days of curing, reaching a maximum of 11.81% and 9.50% for samples after 28 and 56 days of curing, respectively, as compared to standards. At the beginning of the curing process, the short period of 2 to 7 days, it was difficult for bacterial spores to penetrate the materials and to come in contact with reagents that facilitate the carbonate precipitation. *B subtilis* induced the improvement of compressive strength by closing the pores and cracks of mortars due to calcium carbonate precipitation on the surface of bacterial cells. The values of compressive strength from mortars containing sporal suspension from *B. licheniformis* were low, slightly exceeding the values of the standards.

In a water absorption test (Figure 9b), the same tendency of the compressive strength test was shown, with the best results for *Bacillus subtilis*. Generally, the water absorption of mortars with or without bacterial cells decreased with curing time. The effect of introducing the sporal suspension from our *Bacillus subtilis* in mortar mixture was a decrease in water absorption capacity of 11.79% and 10.94% after 28 and 56 days of curing, respectively, as compared to standards. The water absorption values for mortar samples treated with spores from *B. licheniformis* did not differ significantly from those of the untreated specimens. The average decrease compared to standards was 2.64% for all samples analyzed.

## 4. Discussion

The economic and social development of the world is based on the construction industry. Cement, mortars and concrete are building materials that are responsible for environmental pollution, with 10% of total CO_2_ emission [50]. Since there is an increasing interest in developing construction materials through green solutions, there are many studies focused on the attempt to apply the extraordinary ability of microorganisms to precipitate calcium carbonate [50,51,52,53,54]. The pores and cracks of cement represent the pathways through aggressive agents penetrate in matrixes, causing the deterioration and shortening the life cycle. Carbonate could act as a sealant to consolidate the material. There is a need for another type of cement, the cement embedded with bacterial cells which have a positive role in improving the strength of concrete and in the self-healing of cracks.

The main motive of this experimental study was to evidence the positive effect on mortar properties by adding in mortar a calcifying Gram-positive bacterium.

To reach the best-performing bacteria, four bacterial strains belonging to *Bacillus* (3) and *Pseudomonas* (1) genera were cultivated on media supplemented with urea and calcium chloride to sustain the precipitation of calcium carbonate. The results of a qualitative colorimetric test with alizarin red S allowed a preliminary selection, since the positive result exposed by *Bacillus* strains was more evident compared with those from *Pseudomonas* which exhibited a lower level of biomineralization (Figure 1). The optical microscopical investigation of deposits demonstrated the formation of carbonate crystals (Figure 2).

Since the precipitation of calcium carbonate occurred through a ureolytic mechanism, we have tested the capacity of *Bacillus* strains to secrete urease by culturing on solid selective culture media of various composition (Figure 3). On medium M2 with a higher amount of urea (20 g/L), B. subtilis showed a good ureolytic activity, which was followed by *B. licheniformis* and *B. amyloliquefaciens*. Despite our expectations based on literature data [35] that the presence of Ni^2+^ could promote the precipitation of calcium carbonate to some extent, no ureolytic activity was evidenced on medium M3 containing NiCl_2_ (data not shown).

In-depth analyses (FTIR, XRD, TGA) as well as microscopic observations were carried out on deposits from bacterial cultures carried out in agitated flasks on medium with a composition similar to the previously selected one. Crystals of calcium carbonate are highlighted by optical microscopic images, while SEM images presented calcified bacterial cells covered by mucous matrix. Normally, the literature reports the SEM images obtained by gold coating, but it is not possible to obtain high-quality images of biological samples through this procedure [1,55,56].

In general, the FTIR spectra of bacterial cell biomass are relatively differentiated by composition, which implies dependence on the culture age and nutrients quality in the culture medium [48]. The FTIR characteristic signals for CaCO_3_ are as follows: ≈1400 cm^−1^ (ν3—doubly degenerate planar asymmetric stretching), ≈870 cm^−1^ (ν2—out-of-plane bending), and between 700 and 746 cm^−1^ (ν4—doubly degenerate planar bending). The strong vibrational band at 876 cm^−1^ can be assigned to both calcite and vaterite polymorphs, and the differentiation is possible by the band of 700–746 cm^−1^ [32,57]. In our work, the presence of carbonate in samples from all bacteria cultivated in medium with urea and Ca^2+^ was proved by the presence of the stretching band at 1400–1413 cm^−1^ (Figure 6). It can be observed that only for precipitates from cultures on medium with urea and calcium are the amino-related and organic peaks presented around 460 cm^−1^. Since this peak is not present in precipitates from control cultures without urea and calcium, we assume the explication of Šovljanski et al. [49]: that the nutrient medium was not used completely by bacteria after that period of incubation.

Regarding the TGA investigations applied to bacterial spores, it is known that there are two types of water contained in spores, namely absorbed and chemically bounded. The water is located in different places; the absorbed is located in the cortex being associated with cell materials, while chemically bounded water is present in the core and cortex. The temperatures for evaporation are approx. 100 °C for absorbed water and approx. 300 °C for chemically bonded water [58]. In our study, we observed a different thermogravimetric behavior between samples from bacteria cultured on medium with or without urea and calcium chloride, because of samples composition, with or without calcium carbonate embedded in bacterial biomass (Figure 7 and Table 1 and Table 2). Three main weight loss steps were observed in the deposits of samples from bacterial culture on medium with urea and Ca^2+^, *versus* only two steps for the control samples obtained on medium without urea and calcium chloride. The most important result from TGA analysis was the higher values of a residue percentage of samples from bacterial cultivation in media with urea and Ca^2+^ ions compared to those of control samples. This difference was attributed to the precipitation of calcium carbonate under favorable culture conditions, namely the presence of urea and calcium chloride, and ureolytic activity of microbial strain.

In our work, the presence of calcium carbonate precipitated only as calcite was demonstrated by XRD analysis (Figure 8). Calcium carbonate forms three anhydrous polymorphs (calcite, aragonite, and vaterite), two hydrated crystalline phases and various amorphous phases, vaterite and calcite being the most common calcium carbonate polymorphs produced by bacteria. The morphology of the precipitates can largely vary due to several factors such as the composition of the growth medium, temperature and pH, ratio and bacterial species, specific proteins and extracellular polysaccharide secreted by the bacterium [55]. The precipitation of calcite over that of vaterite was observed at *Bacillus licheniformis* related to the dissolved organic carbon released from bacterial extracellular polymeric substances [59]. Several studies reported that mainly calcite as calcium carbonate polymorphism was detected in cultures of *Microbacterium* sp. GM-1 [35] or from *Bacillus subtilis* cultured in the presence of different biological factors (bovine serum albumin, carboxymethyl chitosan and glutamic acid) [60]. On the other hand, there are studies certifying the predominance of calcium carbonate as vaterite. For instance, *B. subtilis* KT343639 could induce amorphous calcium carbonate or polycrystalline vaterite formation according to the concentration of calcium ions in culture medium. It is considered that vaterite has long-term water and thermal stability, and morphology was found to be a function of the rate of precipitation [55].

The end-products from *Sporosarcina pasteurii*, *Bacillus pumilis* and *Bacillus megateriumthat*, which are good producers of urease and carbonic anhydrase, vary in different proportions of two morphologies, calcite and vaterite [61]. The variable composition of proteins secreted by *B. subtilis, Aerobacter aerogenes*, and *Proteus mirabilis* is responsible for the morphology and polymorphs (calcite versus vaterite) in carbonates produced by these bacteria. With the increase in reaction time, the morphologies of the products were changed gradually [17]. Khajani et al. [32] consider that the mechanism that controls polymorph selection and the morphological features of the bacterially precipitated CaCO_3_ is still unclear and controversial. They consider that the prediction and control of carbonate precipitation by bacterium activity is connected to various factors to be analyzed, such as the saturation level, Ca^2+^ and urea concentrations, availability of the organic materials, and the composition and structure of extracellular polymeric substances at the timescale of the experiment.

The efficiency of the supplementation of mortar mixtures with bacterial cells was evaluated by properties such as compressive strength and water absorption. The mechanical properties of mortars are influenced by several factors, such as the composition of the matrix, ratio of components, ratio of water–cement, curing conditions and the incorporation of admixtures and bacterial cells.

The compressive strength and water absorption of specimens cured under tap water and those embedded with bacterial cells and cured under urea and calcium chloride solution are represented as a function of curing time (Figure 9). The compressive strength has a direct relation with water absorption, increasing by decreasing the water absorption. The pores of mortars are formed during the hydration process, when mortar reacts with water from capillary pore spaces to precipitate hydration products.

The supplementation of mortar mixtures with sporal bacteria could change the hydration process, influencing the degree of hydration, speed of the process, evolution of the microscopic morphology and pore structure. According to Wang et al. [62], the process is as follows: during the initial moments, the material pores are still open and the bacterial cells have access to nutrients; then, the environment changed and the microbial cells are confronted with more difficult conditions. Calcium carbonate precipitated on the cell surface and within material pores, and in these conditions, the flow of nutrients becomes more difficult, and the cells survived as resistant endospores.

The presence of bacterial cells from *Bacillus subtilis* produced an enhancement of compressive strength by 11.81% and 9.50%, after 28 and 56 days of curing, respectively, as compared to standards. It was concluded that the increase in compressive strength was due to the microbial precipitation of calcium carbonate, which acts as a filler of material pores. Our results were in agreement with those reported in other studies. Abo-El_Enein et al. [63] obtained a 30% improvement of compressive strength using sporal suspension from *Sporosarcina pasterurii*. Kim et al. [64] prepared mortars with 18.3% higher compressive strength due to the metabolic activity of new ureolytic bacteria isolated from river sediments. In another study, *B. subtilis* incorporated in mortar specimens caused a significant increase in compressive strength by 15.6% and 14.8% for concentrations of 3.3 × 10^−1^ and 3.3 mg/mL for cell walls, respectively, in comparison with the control specimens. The study demonstrated that cell walls are more effective than the live bacteria in binding Ca^2+^ to negative CO_3_^2-^ because of the involvement of a proton pump which competes with Ca^2+^ for binding to the negatively charged cell walls [65].

The mortar samples embedded with bacterial cells and cured in a solution containing urea and calcium chloride created the favorable environment for carbonate precipitation, and we have obtained a decrease in water absorption capacity of 11.79% and 10.94% after 28 and 56 days of curing, respectively, as compared to standards (Figure 9b). The bacteria induced calcium carbonate precipitation on the surface or inside the pores of mortars, reducing the porosity and blocking the capillary pores.

The trend in testing our specimens was found to be similar to those reported in other studies. In an attempt to reduce the cost for wastewater processing in industries, industrial wastewater was used in microbiologically induced calcite precipitation [66]. The organic wastewater procured from industrial food processing units contained microbial nutrients and a relative mass number of microorganisms. In those conditions, the water absorption of bio-cement mortar embedded with cells from *Sporosarcina pasteurii* and *Bacillus sphaericus* was noticed as 14.42% at 28 days curing with 100% organic wastewater. Sujatha et al. [67] reported an indigenous soil bacterium which enhanced by 18% the compressive strength of cement mortars. Using *Bacillus sphaerius* isolated from commercially available cement, the mechanical properties of cement were significantly improved: an increase in compressive strength by 36% and a reduction in water absorption were recorded [68].

## 5. Conclusions

The findings in this study demonstrated that *Bacillus subtilis*, a non-pathogenic bacterium, could be a promising bio-calcifying agent, its cell walls being capable of mediating MICP. The mortars embedded with bacterial cells presented a certain improvement of their mechanical properties, namely compressive strength and water absorption, that, through dedicated studies, can be improved to reach values comparable to those in the literature. Therefore, further research should be carried out to find the optimum dosage of bacterial cells to be added in mortar mixture, since there is a significant effect of cell concentration on compressive strength and water absorption. In addition, the longevity and durability of bacterium in mortar will be evaluated. We consider that the results can be used to develop an experimental protocol for improving the properties of mortars using biogenic bacteria.

## Figures and Tables

**Table 1:** Different media for the cultivation of bacteria.

Culture Medium	Composition (g/L)
Nutrient Broth (NB) medium	1, meat extract; 2, yeast extract; 5, peptone; 5, NaCl; pH = 7.4.
Luria Bertani (LB) medium	10, tryptone; 5, yeast extract; 10, NaCl; pH = 7.0
Tryptic Soy Broth (TSB) medium	30, Scharlau dehydrated powder; 20, peptone; 5, NaCl; 2.5, K_2_HPO_4_; 2.5, dextrose; pH = 7.3 ± 0.2
M1 medium for urease activity	1, peptone; 5, NaCl; 2, KH_2_PO_4_; 0.1, glucose; 2, red phenol; 15, agar; 2, urea
M2 medium for urease activity [35]	1, peptone; 5, NaCl; 2, KH_2_PO_4_; 1, glucose; 0.12, red phenol; 15, agar; 20, urea [35]
M3 medium for urease activity [36]	4, glucose; 1, NH_4_Cl; 3, NaHPO_4_; 0.5, NaCl; 1, KH_2_PO_4_; 44.16 µg NiCl_2_; 20, urea
M4 medium for urease activity	4, glucose; 1, NH_4_Cl; 3, NaHPO_4_; 0.5, NaCl; 1, KH_2_PO_4_.
Medium for carbonate precipitation	3, NB (Nutrient Broth—Scharlau dehydrated power); 3, vinasse; 10, NH_4_Cl; 2.12, NaHCO_3_; pH = 7.0 ± 0.2

**Table 2:** Results of TGA analysis applied to deposit samples from bacterial cultivation on medium with urea and Ca^2+^.

Sample	RT–115 °C		115–178 °C		178–253.5 °C		253.5–550 °C		550–700 °C	Residue
	Wt. Loss (%)	T_max_ (°C)	Wt. Loss (%)	T_max_ (°C)	Wt. Loss (%)	T_max_ (°C)	Wt. Loss (%)	T_max_ (°C)	Wt. Loss (%)	700 °C (%)
*B amyloliquefaciens* (BA)	5.37	66.5	24.36	145.0	5.30	228.6	18.92	347.3	0.27	45.78
*B. licheniformis* (BL)	6.59	63.4	17.60	136.0	6.49	236.1	19.62	349.9	0.26	49.44
*B. subtilis* (BS)	4.73	55.3	23.20	138.1	3.01	214.9	20.16	343.9	0.34	48.56

**Table 3:** Results of TGA analysis applied to deposit samples from bacterial cultivation on medium without urea and Ca^2+^.

Sample	RT–100 °C	100–380 °C		380–550 °C		550–700 °C	Residue
	Wt. Loss (%)	Wt. Loss (%)	T_max_ (°C)	Wt. Loss (%)	T_max_ (°C)	Wt. Loss (%)	700 °C (%)
*B amyloliquefaciens* (MBA)	0.47	68.56	256.6	2.46	399.9	0.65	27.87
*B. licheniformis* (MBL)	0.32	65.41	248.7	2.59	410.1	0.98	30.71
*B. subtilis* (MBS)	0.57	68.59	281.2	2.40	398.1	0.77	27.67

## Data Availability

Not applicable.
